# Emotional regulation or just continuity? Rethinking the emotional function of dreaming in the age of computational sleep science

**DOI:** 10.1093/sleep/zsaf375

**Published:** 2026-04-14

**Authors:** Serena Scarpelli

**Affiliations:** Department of Psychology, Sapienza University of Rome, Rome, Italy

For centuries, dreaming has fascinated humans, and it still remains one of the most interesting puzzles in sleep research today. Although dreams are typically defined as mental activity occurring during sleep and recalled upon awakening, researchers cannot answer many issues about dream experience. Indeed, the question of *why* we dream, and of *whether* this unique form of mentation during sleep *subserves any functional purpose*, remains at the heart of contemporary scientific debate.

Over the last decades, a variety of theoretical models have offered different hypotheses: some attributed to dreaming a functional role in emotional regulation [1], memory consolidation [2], or fear extinction [3], whereas others consider dreams just an epiphenomenon or “residue” of waking experiences without an intrinsic purpose. In this spectrum of interpretations, the continuity hypothesis [4] denies an adaptive function of mental sleep activity, proposing, instead, that dream content merely mirrors waking emotional life. Evidence supporting this view spans different populations and contexts, including recent studies conducted during the COVID-19 pandemic, where individuals with more concerns or health problems during wakefulness reported increased nightmare frequency [5] or research in pregnancy showing that more negative dreams are experienced by women during gestation and new mothers with depressive symptoms [6]. In particular, nightmares provide a compelling lens through which to examine the possible function (if any) of dreaming. Some theoretical models frame nightmares as an opportunity for emotional recalibration and a form of internal exposure that promotes a simulation of potential threats (for a review, see [7]). Others suggest the opposite, since nightmares may be interpreted as a signal of emotional dysregulation, emerging when stress, trauma, or mood disorders overwhelm the system [7].

Within this background, the study by Baber et al. [8] aimed to address a core question in contemporary dream research, namely whether fear experienced in dreams predicts next-morning affect, and whether these effects are modulated by individual characteristics. By focusing on naturalistic dream reports collected over extended periods, the authors contribute to a growing movement in the field: the shift toward large-scale, ecologically valid datasets, and computational linguistic approaches capable of capturing the complexity of dream phenomenology. Firstly, from a methodological perspective, the transition to these approaches marks a significant turning point. Historically, dream research relied heavily on labor-intensive manual coding of relatively small samples of dream reports. More recently, the field has embraced computational tools, such as graph-theoretic metrics, semantic embeddings, and machine-learning techniques, that allow thousands of reports to be analyzed rapidly and systematically [9]. Complementing this line of work, the development of extensive archive of electroencephalographic (EEG) recordings associated to dream collection such as the DREAM database project [10] has provided the large corpora needed to test such tools at scale and to support quantitative content analyses across diverse populations.

Against this methodological backdrop, Baber et al. [8] leverage a validated language model together with Bayesian multilevel modeling, aligning with current trends. Naturalistic dream data vary widely both within and between individuals, and the hierarchical approach used here allows the authors to distinguish daily fluctuations in dream affect from more stable, person-specific traits. With hundreds of participants contributing multiple dream reports, the study avoids one of the most persistent limitations in dream research concerning the insufficient sample size.

Notably, contrary to authors’ expectations, dream fear did not predict reduced negative affect the following morning. Instead, nights marked by greater dream fear were associated with higher next-morning negative affect. More surprisingly, this relationship was amplified among individuals who reported greater use of adaptive emotion-regulation strategies. This counterintuitive result challenges the notion that adaptive regulation skills necessarily buffer individuals from emotional carryover effects, instead suggesting a mere mirroring between dream and waking affect and thus providing support for the continuity hypothesis. One possible interpretation is that individuals with higher regulatory abilities may be more attuned to fluctuations in their internal states and thus more likely to acknowledge negative affect upon waking from a frightening dream. Another possibility proposed by authors is that emotional regulation may operate after the initial surge of negative affect rather than preventing it. In either case, these findings highlight the need to reconsider how regulatory traits interact with dream–wake emotional dynamics.

At the same time, the study revealed another interesting finding regarding the presence of multiple—and opposite—emotions in individuals’ dream experiences: dreams characterized by both fear and joy, as a potential indicator of emotional complexity, were associated with mornings free of negative affect. This aligns with broader work in affective science suggesting that emotional complexity may promote resilience by enabling individuals to experience negative emotions within a broader, more flexible emotional framework. Recent work describing “cathartic dreams,” which dynamically shift from threat to resolution [11], is consistent with this perspective. Though preliminary, the present findings suggest that mixed emotional content may reflect more constructive overnight processing than fear alone. Furthermore, the study demonstrated an association between average dream joy and higher next-day positive affect, supporting the emerging idea that positive dream emotions may play a distinct and beneficial role in waking emotional functioning.

In a broader perspective, the theoretical perspectives most often mentioned in dream research, i.e. those emphasizing fear extinction and those highlighting emotional continuity or affective carryover, need not be mutually exclusive. In fact, emotional recalibration may require the interaction of multiple mechanisms: not only the emotional content of dreams, but also stable dispositional traits and, also, the neurophysiological patterns supporting overnight processing. This underscores the importance of longitudinal designs, which can capture within-person dynamics across nights, and highlights the value of combining linguistic analyses with physiological measures. In this regard, it should be noted that electrophysiological (EEG) patterns such as REM-related theta activity, which has been implicated in emotional memory processing and salience tagging (e.g. [12]), could modulate whether dream emotions facilitate adaptive processing or instead contribute to next-morning negative affect. Likewise, individual differences in personality or trait-level affectivity may shape whether dream fear is transformed, integrated, or maintained across sleep. In this view, integrating dream content with EEG markers and dispositional variables would allow future research to test more mechanistic models of how different emotional processes converge during sleep and ultimately determine whether dreams support or hinder affective recalibration.

Despite these promising insights, one of the main limitations of the study stemming from the fact that the data collection was carried out during the exceptional context of the COVID-19 pandemic, marked by heightened stress and sleep disruption, may have amplified dream negativity and its relationship with morning affect. Furthermore, the absence of objective sleep measures (e.g. polysomnography) prevents examination of whether specific sleep EEG microstructural features and topography moderated the associations observed between dreaming and emotional processes.

Considering all these considerations, the study by Baber et al. (2025) represents an important step forward. Whether dreams regulate emotion, mirror waking concerns, or participate in a more nuanced bidirectional interplay may ultimately depend on the individual, the emotional context, and the complexity of the dream itself.
